# Cinnamic acid, a natural plant compound, exhibits neuroprotection in a mouse model of Sandhoff disease via PPARα

**DOI:** 10.1515/nipt-2023-0027

**Published:** 2024-03-15

**Authors:** Sumita Raha, Ramesh K. Paidi, Debashis Dutta, Kalipada Pahan

**Affiliations:** Department of Neurological Sciences, 2468 Rush University Medical Center, Chicago, IL, USA; Division of Research and Development, Jesse Brown Veterans Affairs Medical Center, Chicago, IL, USA

**Keywords:** Sandhoff disease, cinnamic acid, glial inflammation, GM2 gangliosidosis, PPARα, neuroprotection

## Abstract

Tay-Sachs disease (TSD) and its severe form Sandhoff disease (SD) are autosomal recessive lysosomal storage metabolic disorders, which often result into excessive GM2 ganglioside accumulation predominantly in lysosomes of nerve cells. Although patients with these diseases appear normal at birth, the progressive accumulation of undegraded GM2 gangliosides in neurons leads to early death accompanied by manifestation of motor difficulties and gradual loss of behavioral skills. Unfortunately, there is still no effective treatment available for TSD/SD. The present study highlights the importance of cinnamic acid (CA), a naturally occurring aromatic fatty acid present in a number of plants, in inhibiting the disease process in a transgenic mouse model of SD. Oral administration of CA significantly attenuated glial activation and inflammation and reduced the accumulation of GM2 gangliosides/glycoconjugates in the cerebral cortex of Sandhoff mice. Besides, oral CA also improved behavioral performance and increased the survival of Sandhoff mice. While assessing the mechanism, we found that oral administration of CA increased the level of peroxisome proliferator-activated receptor α (PPARα) in the brain of Sandhoff mice and that oral CA remained unable to reduce glycoconjugates, improve behavior and increase survival in Sandhoff mice lacking PPARα. Our results indicate a beneficial function of CA that utilizes a PPARα-dependent mechanism to halt the progression of SD and thereby increase the longevity of Sandhoff mice.

## Introduction

While Tay-Sachs disease (TSD) is caused due to mutation of *Hexa* gene encoding the α subunit of β-hexosaminidase, the mutation of *Hexb* gene encoding the β subunit of β-hexosaminidase leads to Sandhoff disease (SD), the severe form of TSD. Both TSD and SD are progressive neurodegenerative lysosomal storage disorders characterized by excessive accumulation of GM2 ganglioside, predominantly in the central nervous system [1, 2]. These neurodegenerative diseases are often characterized by acute neurodegeneration preceded by activation of microglia and astrocytes, leading to enhanced neuroinflammation [3, 4]. Unfortunately, there is still no treatment available for these diseases.

Gangliosides are the major glycosphingolipids or glycoconjugates of the neuronal cell membrane, ensuring normal cellular activity [5, 6]. The enzyme beta hexosaminidase A (Hex A), is responsible for GM2 ganglioside degradation [7]. Defective GM2 ganglioside production due to deficiency of Hex A enzyme, progressive accumulation of GM2 in neuronal cells and subsequent neurodegeneration are pathological hallmarks of TSD [2, 6]. The progressive neurodegeneration seen in Tay-Sachs disease depends upon the rate and degree of GM2 ganglioside deposits, which in turn is inversely proportional & determined by the level of functional β-hexosaminidase A present in the body [7, 8]. The GM2 ganglioside accumulation, therefore, leads to several cytotoxic effects that take place mainly in neurons, causing neuronal death [9].

Glial activation including astrogliosis and microgliosis and other associated neuroinflammation participate in the pathogenesis of several neurodegenerative disorders [4, 10], [11], [12]. Astrocytes constitute the majority of the glial cells and play a crucial role in brain homeostasis. Potential dysfunctions of astrocytes and astrogliosis have been identified as a primary cause of neurodegenerative diseases [13, 14]. Microglia on the other hand serves as scavenging cells in the CNS for the degradation of extracellular debris and protein aggregates via phagocytosis [15], [16], [17]. Numerous reports have shown that the accumulation of GM1 and GM2 gangliosides in the CNS led to microgliosis and astrogliosis, and the extent of inflammation is associated with enhanced levels of ganglioside accumulation [18, 19]. Therefore, agents capable of inhibiting glial activation and inflammation in ganglioside pathogenesis might render neuroprotection in TSD and SD.

Cinnamic acid (CA), found in many plants as deaminated *product* of phenylalanine, is basically an organic compound. This unsaturated carboxylic acid is one of the many pigments responsible for the distinctive color of balsam tree sap. CA is used in different industries. It also has a long history of human use as a component of *plant*-derived scents. While it is found to be beneficial in treating diabetes [20], according to Liu and colleagues [21], CA can also induce cytostasis and reverse malignant properties of human tumor cells. Earlier we have shown that oral CA is capable of reducing plaques in an animal model of Alzheimer’s disease [22] and protecting dopaminergic neurons in an animal model of Parkinson’s disease [22]. Here we examined the effect of CA on inflammation and overall glycoconjugate induced pathology in a mouse model of SD and observed that oral administration of CA could reduce glial inflammation and lower glycoconjugates from the cerebral cortex of Sandhoff mice. Our previous studies reported CA as an agonist of peroxisome proliferator-activated receptor α (PPARα) [22]. Therefore, we investigated its role in CA-mediated neuroprotection and found that CA was unable to reduce SD pathology and increase longevity in Sandhoff mice lacking PPARα. Our results suggest that oral CA may have therapeutic significance for increasing the longevity in SD.

## Materials and methods

### Reagents

Trans-cinnamic acid was obtained from Sigma Aldrich (C80857) Anti-Iba1 antibodies were purchased from Abcam, (Cambridge, MA). Anti-GFAP antibody was procured from DAKO, whereas anti-iNOS antibody was bought from BD Bioscience (San Jose, CA). Details about the antibodies are mentioned in Table 1.

**Table 1:** List of antibodies used.

Target and antibody	Source	Catalog no.	Application/dilution	Host
GFAP	Dako	IS524	WB/1:1000; IHC/1:500	Rabbit
Iba1	AbCam	ab5076	WB/1:1000 IHC/1:1000	Goat
iNOS	BD bioscience	610329	WB/1:1000 IHC/1:200	Mouse
β Actin	AbCam	ab6276	WB/1:1000	Mouse
NeuN	ThermoFisher scientific	702022	IHC/1:1000	Rabbit
PPARα	Santa Cruz	sc-398394	WB/1:1000; IHC/1:250	Mouse

WB, Western blot; ICC, IHC, immunohistochemistry.

### Animals

Sandhoff (B6;129S4-Hexb^tm1Rlp^/J) mice lacking the *Hexb* gene was purchased from Jackson Laboratories. Experimental mice were housed under standard conditions with access to food and water *ad libitum*. Mice were bred and screened by genotyping. Sandhoff mice was also bred with PPARα knock out mice to generate Sandhoff^ΔPPARα^ mice or SD^ΔPPARα^ mice. Animal maintenance and experiments were performed in accordance with the National Institutes of Health guidelines and were approved by the Institutional Animal Care and Use committee of the Rush University Medical Center (Chicago, IL).

### CA treatment

CA was solubilized in 0.1 % methyl cellulose solution. Sandhoff mice (3-months-old) were treated with CA at doses of 25 and 50 mg/kg/day via gavage. Each mouse was fed with 100 µL of CA solution by oral gavage daily for 60 days. In general, any animal experiment is justified with 99 % confidence interval that generates **p**=0.99 and (**1−p**)=(1–0.99)=0.01; **ε** is the margin of error =0.05. Based on these values, the resultant sample size is: n=1,282 × 0.99(1−0.99)0.052=1,282 × 0.99 × 0.01 × 0.052=0.016 × 0.0025=6.48. Therefore, six mice (n=6) were used in each group.

### Perfusion of mice

Mice were perfused transcardially with PBS followed by keeping half brain at −80 C for Western blot and half at room temperature in paraformaldehyde solution for immunohistochemistry.

### Western blotting

Western blotting was performed as described in our earlier studies [14, 23]. Equal amounts of proteins were electrophoresed in 10 % or 12 % SDS-PAGE and transferred onto a nitrocellulose membrane. The blot was probed with primary antibodies overnight at 4 °C. The following are the primary antibodies used in this study: anti-iNOS (1:1000, BD Biosciences), anti-Iba1 (1:1000, Abcam), anti-GFAP (1:1000, Santa Cruz Biotechnology, Dallas, TX, USA) and anti-β-actin (1:5000, Abcam). Details about the antibodies are provided in Table 1. Following the overnight incubation, primary antibodies were removed, the blots were washed with phosphate buffer saline containing 0.1 % Tween-20 (PBST) and corresponding infrared fluorophore-tagged secondary antibodies (1:10,000, Jackson Immuno-Research) were added at room temperature. The blots were then incubated with secondary antibodies for 1 h. Later, blots were scanned with an Odyssey infrared scanner (Li-COR, Lincoln, NE, USA). Band intensities were quantified using the ImageJ software (NIH, Bethesda, MD, USA).

### Immunohistochemistry

Immunohistochemistry was performed as described earlier [14, 15]. Briefly, coronal sections (30 μm thickness) were cut from the forebrain containing striatum and cerebral cortex. Sections were blocked with 3 % normal horse serum and 2 % BSA made in PBST containing 0.5 % Triton X-100 (Sigma-Aldrich) for 1 h. Then the sections were kept in primary antibodies (Table 1) and incubated at 4 °C temperature overnight under shaking conditions. Next day, the samples were washed with PBST for at least three times, 10 min each, and further incubated with Cy2- or Cy5-labeled secondary antibodies (all 1:500; Jackson Immuno-Research) for 1 h under similar shaking conditions. Following several washes with PBST, sections were incubated for 5 min with 4′,6-diamidino-2-phenylindole (DAPI, 1:10,000; Sigma-Aldrich) for immunofluorescence. Mean fluorescence intensity (MFI) was measured using Fiji (ImageJ2) [24, 25].

### PAS and H&E staining

Periodic acid-Schiff (PAS) staining was carried out with PAS stain kit from Abcam (ab150680) as instructed by the manufacturer. PAS-stained sections were counterstained with haematoxylin, dehydrated, cleared with a series of solutions of increasing concentrations of ethanol and xylene and mounted in DPX (dibutyl phthalate xylene, British drug houses).

### Open field test

Open field test was performed to monitor the locomotor abilities of the animals on a horizontal plane. Movement associated parameters were captured with a camera linked to Noldus system and EthoVision XT software (Netherlands). The instrument records the overall movement abilities of the animals such as total distance moved, velocity, moving time, resting time, center time, and frequencies of movement. Before recording the movement, all experimental mice were placed inside the open field arena for 10 min daily for 2 consecutive days for training and recording baseline values. Next day, animals were given rest and the following day each mouse was gently placed in the middle of the open field arena. After releasing the animal, data acquisition was started by the software for the next 5 min [26], [27], [28].

### Rotarod

Animals were placed on the rotating rod against the direction of rotation. The machine was set to run at a gradual increasing speed of 4–40 rpm. The time for spending on the rotating rod was recorded and the experiment was ended once the animal slips from the rod to the base of the instrument [15, 29].

### Gait analyses

Mice were acclimatized by making them walk on a slanting platform for consecutive two days. Each mouse was given five trials each day to walk on the platform to the ascending direction. After a gap of one day, the experiment was performed. The gangway was covered with a long white paper and the limbs of the animals were painted with non-toxic black colored ink to get the impression of the footprints of each animal. Following the experiment, based on the footprints different gait parameters such as stride length, stride width, foot length and toe spread were measured. If any animal stopped or started walking in reverse direction, experiment for that animal was repeated [16, 30].

### Survival assay

Another cohort of mice were sacrificed at end-point to assess survival analysis. All mice were followed daily for 6 months to record survival. Survival time reflects the time required for the animals to reach parameters of measurable endpoints such as reduced gait & motor movements and paralysis of fore and hind limbs. Survival data were plotted on a Kaplan–Meier method, and different groups were compared using the Log-rank (Mantel–Cox) test (GraphPad Prism Software v.9.0).

### Statistical analyses

Statistics were performed using GraphPad Prism version 9.0. One-way ANOVA followed by Tukey’s multiple comparison test was performed for analyzing statistical significance among multiple samples, whereas unpaired two-tailed t-test was performed to compare two samples. Values are expressed as mean ± S.E.M. The criterion for statistical significance was p<0.05.

## Results

### Oral administration of CA reduces astroglial activation in Sandhoff mice

**Figure 1:**
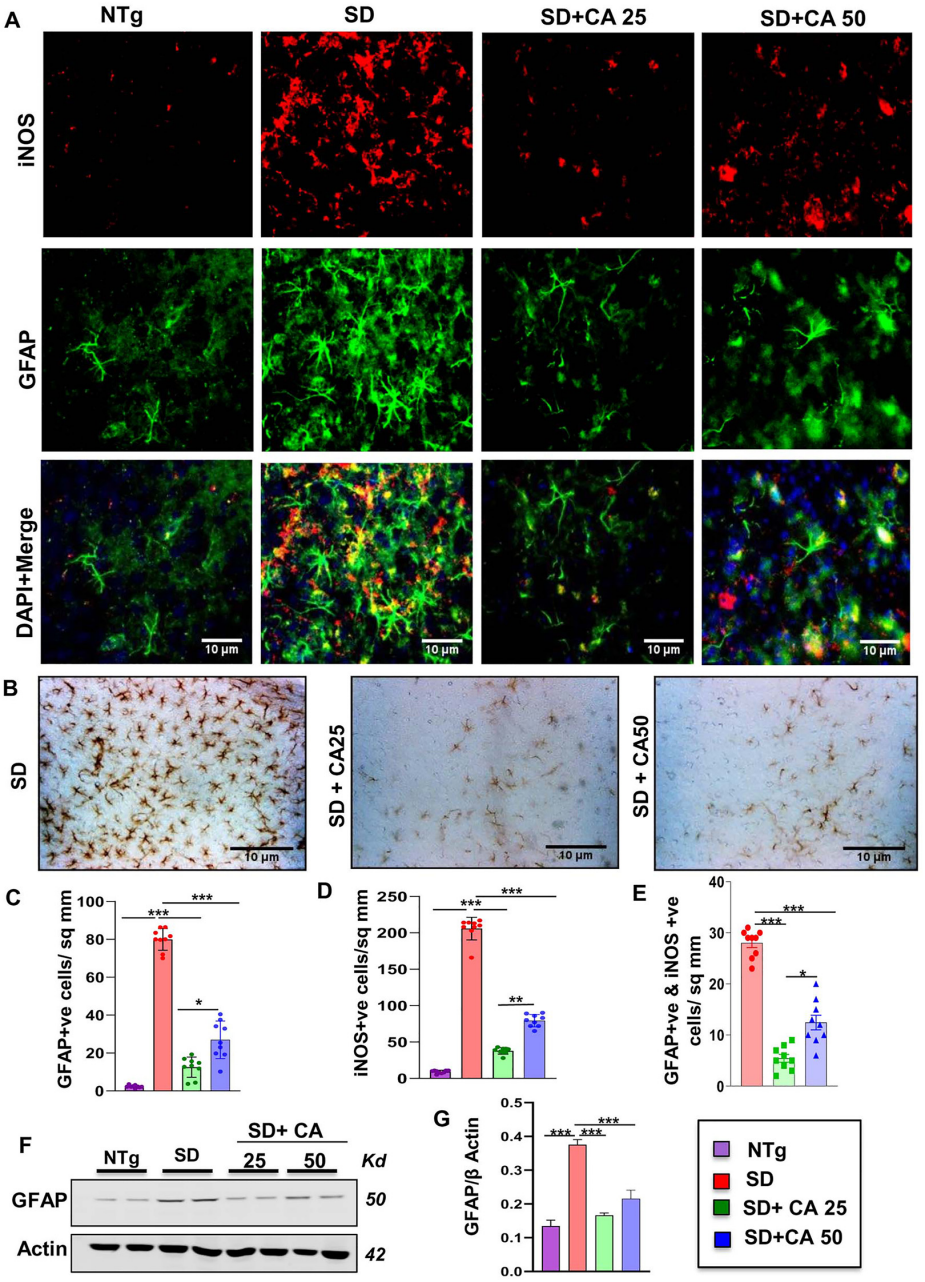
Oral administration of CA alleviates the activation of astrocytes in the cerebral cortex of Sandhoff mice. Three-months-old SD mice (n=6/group) were treated with two doses of CA (25 and 50 mg/kg/day) solubilized in 100 µL 0.5 % methylcellulose, via oral gavage. Therefore, control SD mice received 100 µL 0.5 % methylcellulose as vehicle. After 2 months of daily treatment, mice were sacrificed followed by keeping half brain for Western blot and half for immunohistochemistry. Age-matched non transgenic mice (n=6/group) were used as control. Astroglial activation was monitored in the cerebral cortex sections for GFAP and iNOS through double-label immunofluorescence (A) followed by diaminobenzidine staining of cerebral cortex sections stained for GFAP showing astrogliosis with GFAP-positive astrocytes (B) quantification of iNOS +ve (C) and GFAP +ve cells (D) and iNOS +ve, GFAP +ve cells (E) in 9 sections from a total of 3 mice per group with image J software. Cerebral cortex homogenates (n=4 per group) were subjected to immunoblot analysis for GFAP using β actin as loading control (E). Densitometric analysis of relative iNOS (iNOS/actin) (F) and GFAP (GFAP/actin) (G) levels with respect to non-transgenic was measured for 4 mice per group with ImageJ. All data represent mean ± SEM. Statistical analyses were performed by one-way ANOVA followed by Dunnett’s multiple comparison test; ^*^p<0.05; ^**^p<0.01; ^***^p<0.001. GFAP, glial fibrillary acidic protein; iNOS, inducible nitric oxide synthase.

Although astrocytes constituting most of the glial cells play a critical role in brain homeostasis, potential dysfunctions of astrocytes and astrogliosis have been identified as an important cause of many neurodegenerative diseases including TSD and SD [31, 32]. As evidenced by immunostaining of astrocytes by its marker glial fibrillary acidic protein (GFAP), astroglial activation was found to be greater in the cerebral cortex region of SD mice than non-transgenic (NTg) mice (Figure 1A and C). Furthermore, astroglial inflammation was substantiated by the elevated expression and colocalization of iNOS in the activated astrocytes of vehicle treated SD brains as compared to the NTg mice (Figure 1A, D and E). However, oral treatment with two different doses of CA viz. 25 mg/kg body weight (CA25) and 50 mg/kg body weight (CA50) significantly reduced astrogliosis and astroglial expression of iNOS in SD brain (Figure 1A and C–E). While both the doses showed a substantial reduction in the total number of astrocytes, the lower dose (CA25) was more effective than the higher one (CA50) in reducing the total number of astrocytes in the cerebral cortex SD mice (Figure 1A and C). This was further validated with DAB immunohistochemistry where the GFAP immunoreactivity was substantially higher in vehicle treated SD mice than NTg mice and the treatment with both CA25 and CA50 reduced the number of astrocytes (Figure 1B). Moreover, immunoblot analysis confirmed a significant increase in the protein expression of GFAP in the cerebral cortex of SD mice as compared to that of NTg mice and suppression of GFAP by oral cinnamic acid treatment (Figure 1F and G). For raw Western blots, please see Figure S1.

### Oral administration of CA inhibits microglial activation in the cerebral cortex of Sandhoff mice

**Figure 2:**
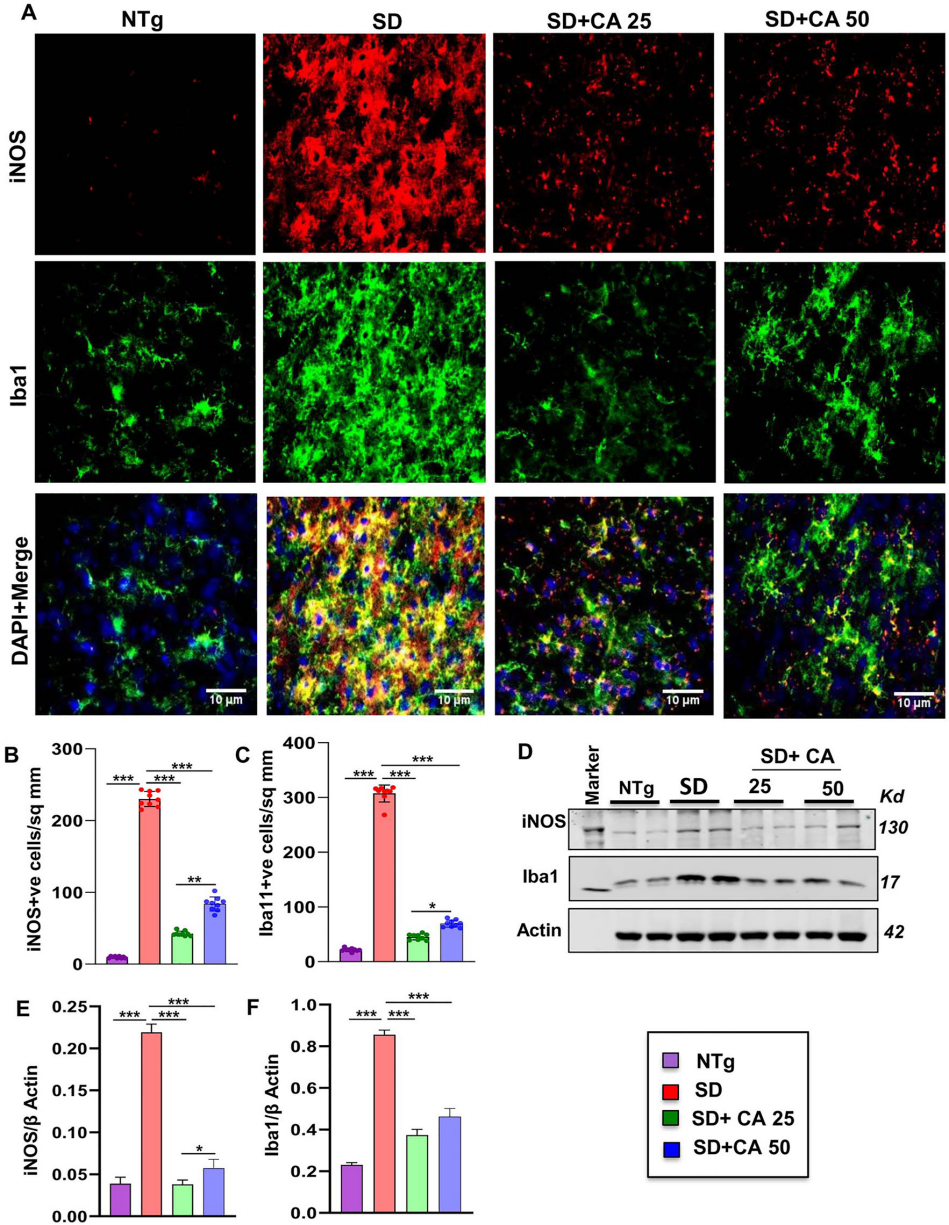
Oral administration of CA inhibits microglial activation *in vivo* in the cerebral cortex of Sandhoff mice. Three-months-old SD mice (n=6/group) were treated with two doses of CA (25 and 50 mg/kg/day) solubilized in 100 µL 0.5 % methylcellulose, via oral gavage. Therefore, control SD mice received 100 µL 0.5 % methylcellulose as vehicle. After 2 months of daily treatment, mice were sacrificed followed by keeping half brain for Western blot and half for immunohistochemistry. Age matched non transgenic mice (n=6/group) were used as control. Microglial activation was monitored in brain (cerebral cortex) sections by double-labelled immunofluorescence for Iba1 and iNOS (A) followed by quantification of iNOS +ve (B) and Iba1 +ve (C) cells. Results represent counting of three different sections from three different mice (n=3) per group with image J software. Cerebral cortex homogenates (n=4 per group) were subjected to immunoblot analysis for Iba1 and iNOS using β actin as loading control (D). Densitometric analysis of relative iNOS (iNOS/actin) (E) and Iba1 (Iba1/actin) (F) levels with respect to non-transgenic was measured. All data represent mean ± SEM. Statistical analyses were performed by one-way ANOVA followed by Dunnett’s multiple comparison test; ^*^p<0.05; ^**^p<0.01; ^***^p<0.001. Iba1, ionized calcium binding adapter molecule 1.

Microglial activation is the key regulator of inflammatory responses in the CNS and therefore, neurodegenerative disorders are often characterized by proliferation and activation of microglia. We investigated the effect of oral CA on microgliosis and inflammation. Immunostaining of microglia specific marker, ionized calcium binding adaptor molecule 1 (Iba1), exhibited that the vehicle treated Sandhoff transgenic mice had significantly higher number of microglia in the cerebral cortex when compared to NTg mice (Figure 2A and C). Accordingly, the expression of nitrosative stress marker inducible nitric oxide synthase (iNOS) was also found to be significantly upregulated in Iba1+ve microglia in the cerebral cortex of Sandhoff mice in comparison to NTg mice (Figure 2A and B). The enhanced protein expression of Iba1 and iNOS was further confirmed by immunoblot analysis from these brain tissues (Figure 2D–F). For raw Western blots, please see Figure S2. Similar to the reduction in astroglial activation, microglial number and expression of iNOS also decreased after CA administration in Sandhoff mice (Figure 2). Again, CA25 was more effective than CA50 in reducing microglial inflammation in the cerebral cortex of Sandhoff mice (Figure 2). These results suggest that oral CA is capable of reducing microglial inflammation *in vivo* in the brain of Sandhoff mice.

### Oral CA attenuates neuronal apoptosis and lowers the cerebral glycoconjugate load in Sandhoff mice

**Figure 3:**
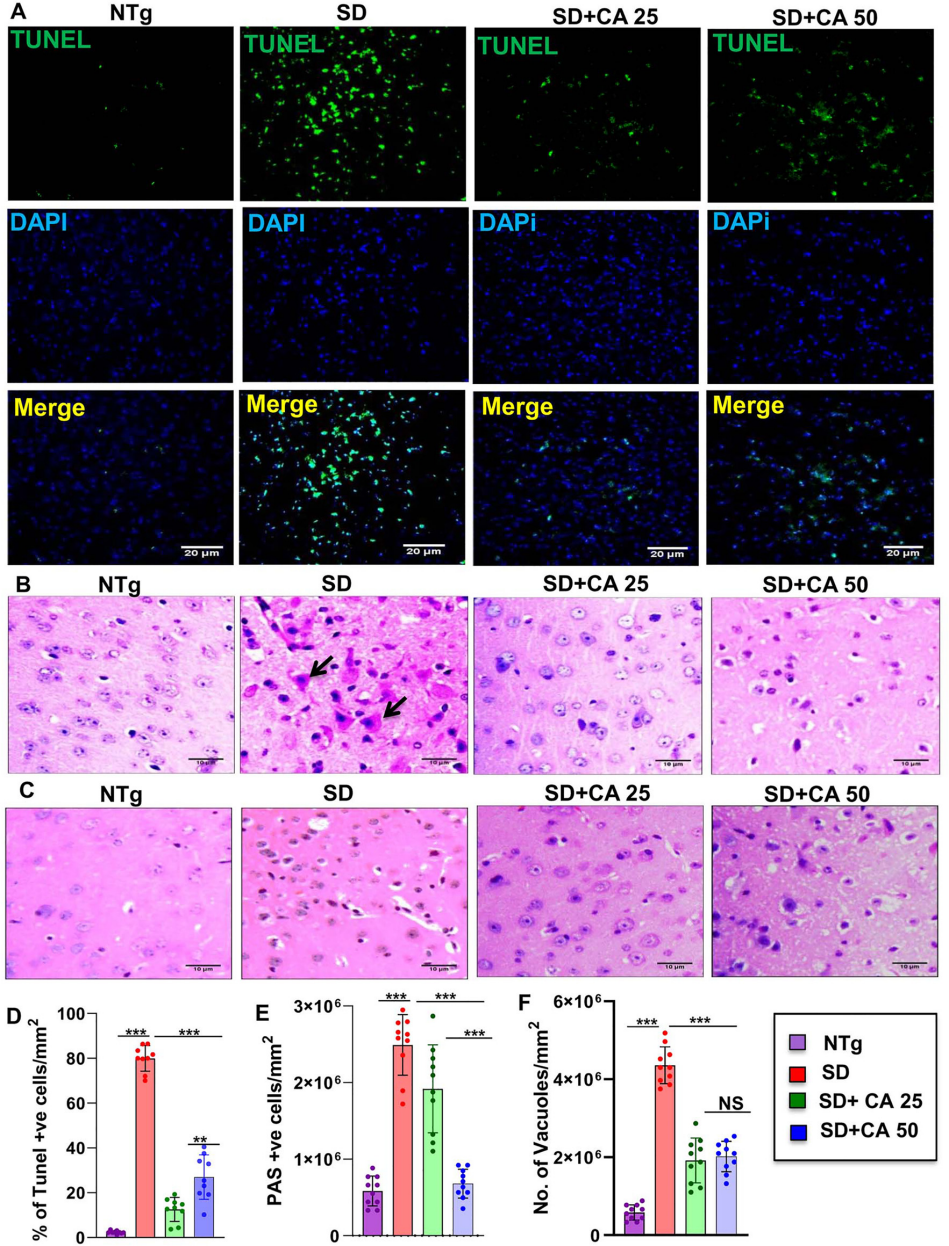
Treatment with CA attenuates neuronal apoptosis and reduces glycoconjugates in Sandhoff mice. Three-months-old SD mice (n=6/group) were treated with two doses of CA (25 and 50 mg/kg/day) solubilized in 100 µL 0.5 % methylcellulose via oral gavage. Therefore, control TS mice received 100 µL 0.5 % methylcellulose as vehicle. After 2 months of treatment, cortical sections were stained for TUNEL (A). The TUNEL (+ve) cells were expressed as % age of total cells per square mm (B). Paraffin embedded cerebral cortex sections were subjected to periodic acid-Schiff (PAS) stain for analyzing glycolipids (C). The glycoconjugate material-stained magenta is shown by arrow and quantified for PAS +ve cells per mm square (D). Paraffin embedded sections were also subjected to H & E staining as characterized by large vacuoles (thick arrows), pyknotic nuclei and swollen neuron in TS mice (E). The number of vacuoles were quantified per mm square (F). Results represent counting of 9–10 different sections from a total of 3 mice (n=3) per group. All data represents mean ± SEM. All statistical analysis was performed by one-way ANOVA followed by Dunnett’s multiple comparison test. ^*^p<0.05; ^***^p<0.001.

Apoptosis was detected by *in situ* terminal deoxynucleotidyl transferase-mediated dUTP nick end labeling (TUNEL) method and we found induction of apoptosis in the cerebral cortex of 5-months-old Sandhoff mice as compared to age matched NTg mice (Figure 3A). Counting of TUNEL-positive bodies confirmed the presence of significantly higher number of apoptotic neurons in the cerebral cortex of Sandhoff mice than that of age matched NTg mice (Figure 3D). However, oral treatment with CA significantly reduced neuronal apoptosis in the cerebral cortex of Sandhoff mice (Figure 3A and D).

We also used routine hematoxylin and eosin (H&E) stained sections to evaluate the tissues of the cerebral cortex region of the brain and then added periodic acid-Schiff (PAS) for characterizing intricate details. It has been reported that pathological amounts of GM2 ganglioside and related glycoconjugates are found to be elevated as early as in fetal life, appearing to increase linearly over time [33]. In the present investigation, we evaluated the cerebral cortex region of 5-months-old Sandhoff mice that was stained with periodic acid-Schiff reagent (PAS) to detect stored glycoconjugates, a hallmark of GM2 gangliosides [2, 6]. As expected, we observed greater number of PAS-positive granules in the cerebral cortex of Sandhoff mice in comparison to NTg mice (Figure 3B). PAS-positive granules in Sandhoff mice also revealed widely dispersed staining through immunohistochemistry, which was mostly cytoplasmic (Figure 3B). Besides, Sandhoff mice also had substantially increased amounts of larger and paler staining granular material in the perinuclear cytoplasm of numerous large and medium size neurons as compared to NTg mice (Figure 3B and E). However, oral treatment with CA significantly reduced PAS positive granules with lower quantities of stained granular material present in smaller amounts and in fewer neurons (Figure 3B). The glycoconjugate material-stained magenta are shown by arrow and quantified for PAS +ve granules per mm square (Figure 3B and E). Sandhoff mice showed widely dispersed swollen dystrophic axons, neurites, and spheroids in multiple regions of the tissue as compared to NTg (Figure 3B). Besides, we performed H&E staining, which revealed the presence of severe vacuolation in Sandhoff mice when compared to age-matched NTg mice (Figure 3C and F). In marked contrast, the pathological findings observed in CA-treated Sandhoff mice displayed reduced swollen dystrophic axons, neurites, and significant reduction in the number of vacuoles (Figure 3C and F). Therefore, oral cinnamic acid is capable of clearing the glycoconjugate pathology and restoring apparent normal histology.

### Oral administration of CA improves gait, reduces motor deficits, and increases survival in Sandhoff mice

**Figure 4:**
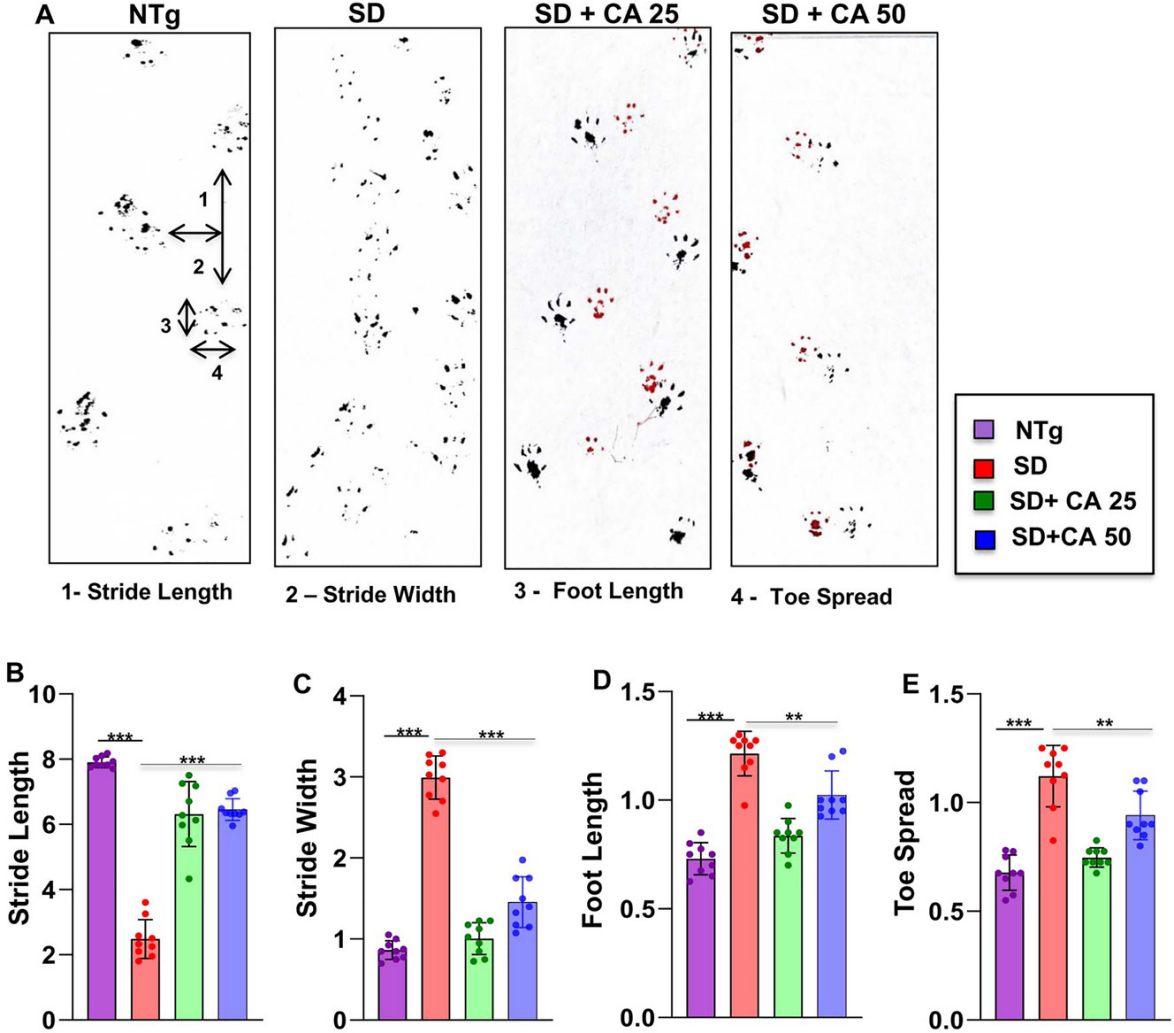
Oral gavage of CA improves gait in Sandhoff mice. Three-months-old SD mice (n=6/group) were treated orally with two doses of CA (25 and 50 mg/kg/day) solubilized in 100 µL 0.5 % methylcellulose via gavage. Control SD mice received 100 µL 0.5 % methylcellulose as vehicle. After 2 months of treatment, mice painted with different colors on the front (red) and hind paws (black) were allowed to walk through a clear tunnel. After pre training sessions, the footprints were measured on a white paper on the runway floor. Footprints were measured for stride length (A, B), stride width (A, C), foot length (A, D) and toe spread (A, E). Data represents mean ± SEM. Statistical analysis was performed by one-way ANOVA followed by Dunnett’s multiple comparison test. ^**^p<0.01; ^***^p<0.001.

The neuroprotective effect of CA treatment against Sandhoff-associated brain pathology necessitated evaluation of the behavioral parameters of the experimental mice. In the present study, our focus was on the cerebral cortex, the regions that are known to regulate motor coordination and movement. For investigating the motor coordination in more details, we conducted footprint analysis for measuring gait. While the stride length of Sandhoff mice was significantly lower than the NTg animals (Figure 4A and B), the toe spread increased due to the disease pathology (Figure 4A and E). This indicates that Sandhoff mice take longer time to cross the gangway due to the compromised movement capability. We also measured stride width (Figure 4A and C) and foot length (Figure 4A and D) of the experimental animals. There was significant increase in the stride width and foot length of the Sandhoff mice as compared to the NTg (Figure 4A, C and D). On the other hand, after CA treatment all parameters including stride length, toe spread, stride width, and foot length of Sandhoff mice significantly showed values that are in tune more towards the values of NTg mice, thus indicating an overall improvement in motor skills (Figure 4A–E).

**Figure 5:**
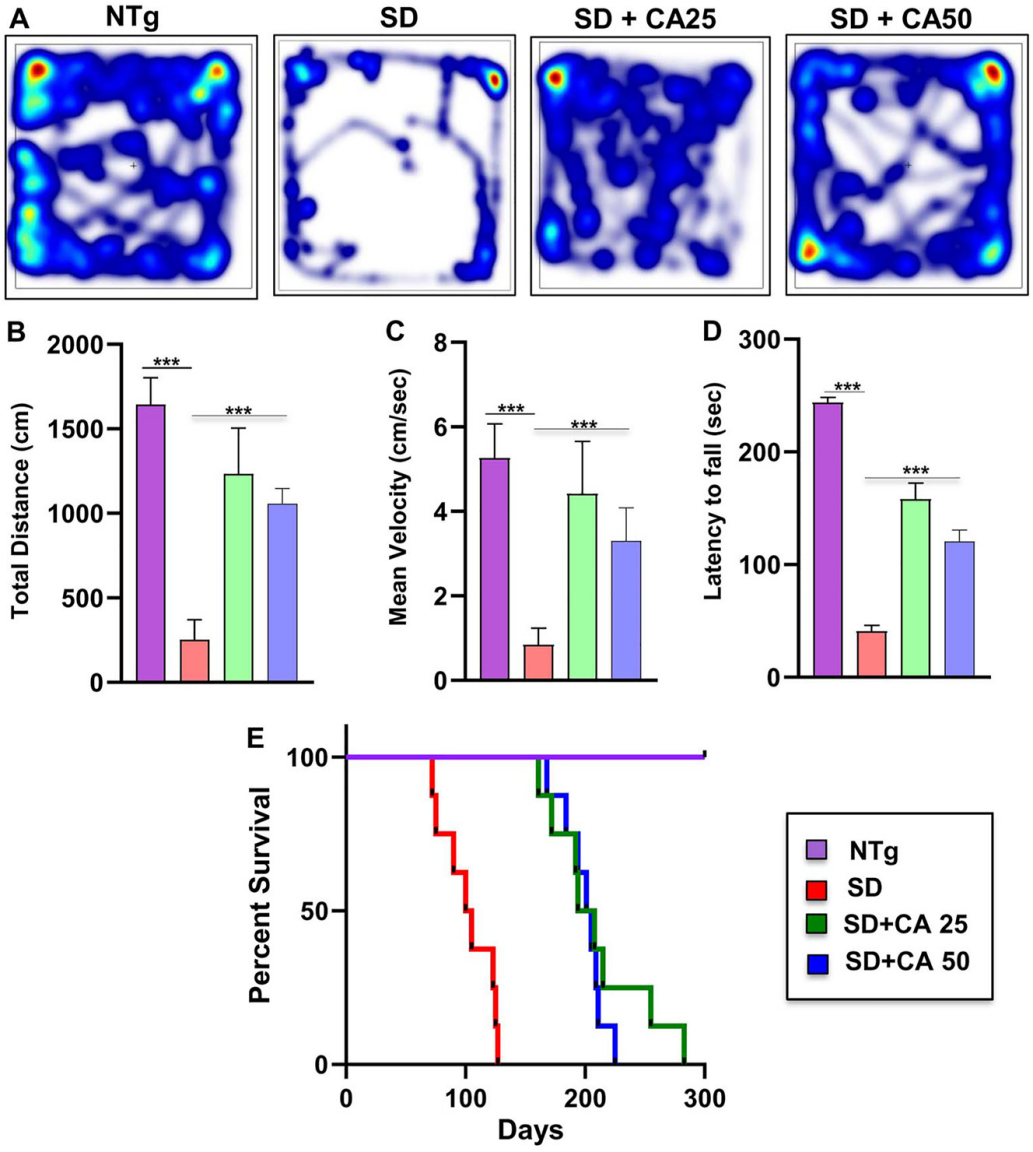
Oral gavage of CA alleviates motor deficits and increases survival in Sandhoff mice. Three-months-old SD mice (n=6/group) were treated orally with two doses of CA (25 and 50 mg/kg/day) solubilized in 100 µL 0.5 % methylcellulose via gavage. Control SD mice received 100 µL 0.5 % methylcellulose as vehicle. After 2 months of treatment, horizontal locomotor activities of experimental animals were monitored in the open field arena by the Noldus software (A, heatmap; B, total distance; C, mean velocity). Latency of each mouse to fall on the base of a rotarod was monitored (D). Kaplan–Meier survival analysis (n=8) (E). Data represent mean ± SEM. Statistical analysis was performed by one-way ANOVA followed by Dunnett’s multiple comparison test; ^***^p<0.001.

Besides we also evaluated the motor behavior performance of the animals. As expected, Sandhoff mice showed extremely poor performance in the open field test, as shown by the reduction in the movement parameters such as distance moved (Figure 5A and B) and velocity of movement (Figure 5A and C) in the arena when compared to the NTg mice. In addition, the coordination of feet movement on rotarod was extremely impaired in Sandhoff mice as compared to NTg mice as evident from the latency to fall (Figure 5D). However, Sandhoff mice administered with CA exhibited significant improved performance in all the behavioral experiments as evident from distance moved and velocity in the open field arena and greater latency in the rotarod.

In general, SD is very fatal in which children with SD die within few years. Similarly, Sandhoff mice also died within 100–120 days. Survival curves for each treatment group were estimated using the Kaplan–Meier method. Oral administration of CA had a marked effect on the life span of Sandhoff mice (Figure 5E). In this case as well, CA25 was more effective than CA50 in increasing the survival of Sandhoff mice (Figure 5E). While CA25-treated Sandhoff mice lived up to 270 days, CA50-treated Sandhoff mice were alive until 210 days (Figure 5E).

### Oral administration of CA upregulates PPARα *in vivo* in the cerebral cortex of Sandhoff mice

**Figure 6:**
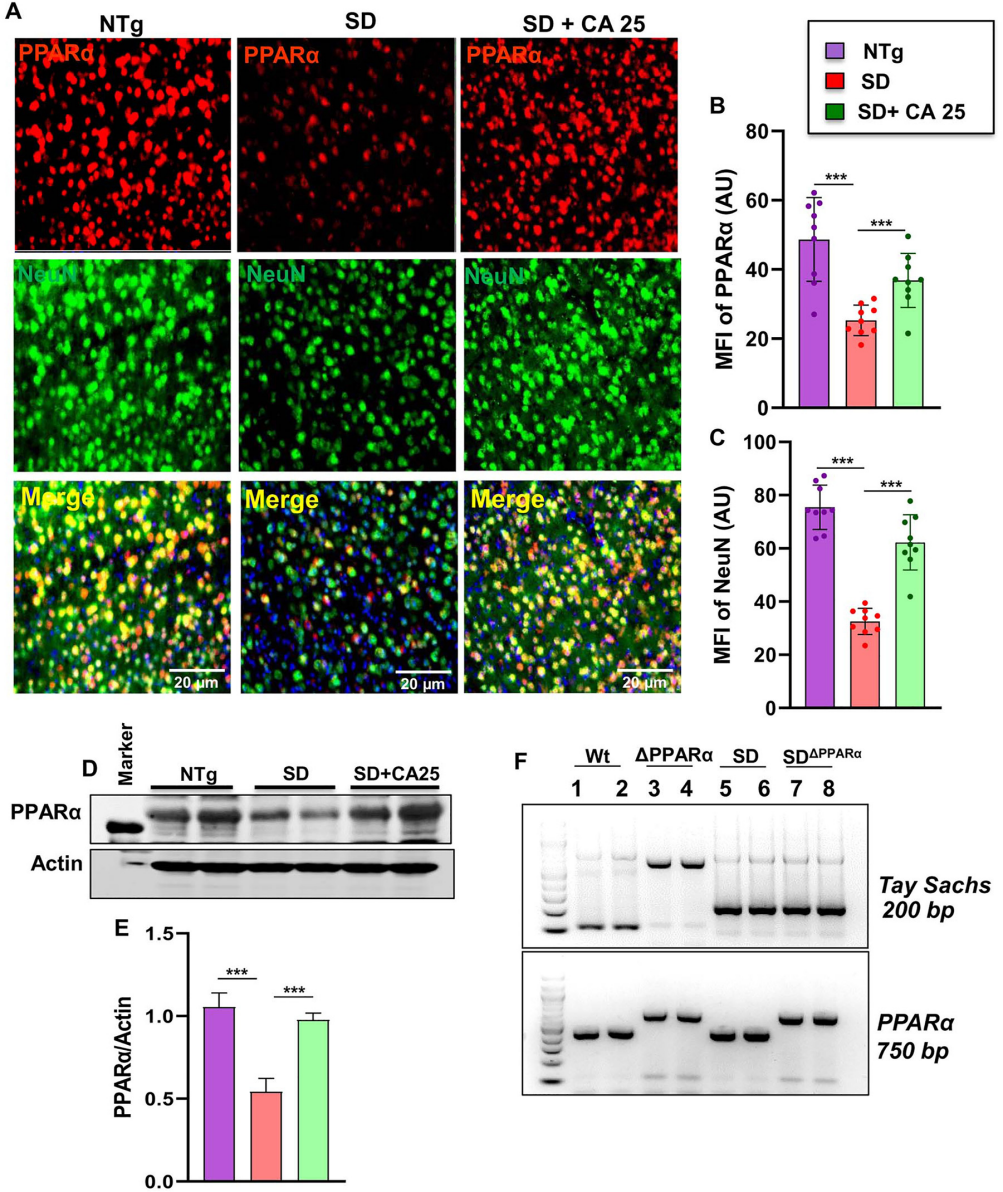
Status of PPARα in the brain of Sandhoff mice. Three-months-old SD mice (n=4/group) were treated orally with CA (25 mg/kg/day) solubilized in 100 µL 0.5 % methylcellulose via gavage. Control SD mice received 100 µL 0.5 % methylcellulose as vehicle. After 2 months of treatment, mice were sacrificed followed by keeping half brain for Western blot and half for immunohistochemistry. Cerebral cortex sections were double-labeled for PPARα (red) and NeuN (green) (A). MFI of PPARα (B) and NeuN (C) from nine images from a total of 4 mice per group. Western blot of PPARα in the cerebral cortex of 4 mice per group (D) followed by densitometric quantification (E). SD mice were bred with PPARα knock out mice to generate SD^ΔPPARα^ lines (F, genotyping). Data represents mean ± SEM. Statistical analysis was performed by one-way ANOVA followed by Dunnett’s multiple comparison test. ^***^p<0.001.

Next, we investigated mechanism by which CA exhibited neuroprotection in Sandhoff mice. Studies from our lab have demonstrated that peroxisome proliferator-activated receptor α (PPARα) acts as an important regulator of many neurodegenerative diseases [24, 34], [35], [36], [37] and that CA binds to the ligand-binding domain of PPARα [22]. We therefore wanted to examine the role of PPARα in CA25-mediated neuroprotection in Sandhoff mice. At first, we checked the status of PPARα in the cerebral cortex of CA25-treated and untreated Sandhoff mice. The cerebral cortex region was double labelled for PPARα and NeuN (Figure 6A–C). As reported earlier, NeuN-positive neurons in the cerebral cortex of NTg mice expressed PPARα (Figure 6A). We found decrease in both PPARα (Figure 6A and B) and NeuN (Figure 6A and C) in the cerebral cortex of Sandhoff mice when compared to the NTg group. However, significant increase in PPARα (Figure 6A and B) and NeuN (Figure 6A and C) was observed in the cerebral cortex of Sandhoff mice treated with CA25 (Figure 6A–C). This was further validated by Western blot, which also showed significant reduction of PPARα in cerebral cortex of Sandhoff mice compared to NTg mice (Figure 6D and E). Again, oral CA25 led to substantial increase in the levels of PPARα in the cerebral cortex of Sandhoff mice (Figure 6D and E). For raw Western blots, please see Figure S3.

Our finding that CA25 could increase the level of PPARα led us to investigate the hypothesis that PPARα activation could be the underlying mechanism by which CA exhibited glycoconjugate lowering effects in Sandhoff mice. We, therefore, generated Sandhoff^ΔPPARα^ (Sandhoff mice lacking PPARα) mice by breeding Sandhoff mice with PPARα null mice (Figure 6F).

### Oral CA mitigates glycoconjugates in Sandhoff mice via PPARα

**Figure 7:**
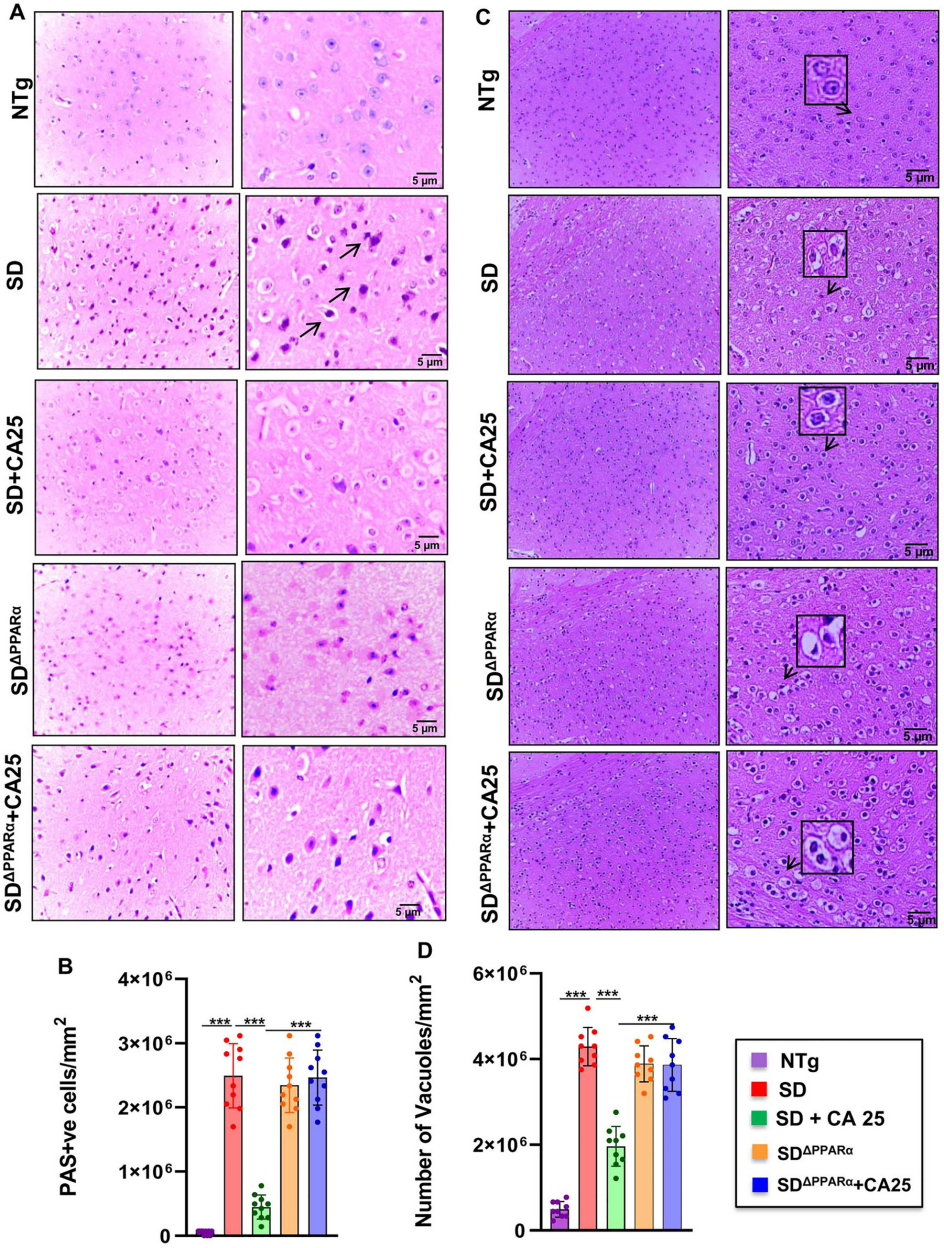
Oral CA reduces glycoconjugate storage in Sandhoff mice via PPARα. Three-months-old SD mice (n=6/group) were treated with CA (25 mg/kg/day) solubilized in 100 µL 0.5 % methylcellulose via oral gavage. Therefore, control SD mice received 100 µL 0.5 % methylcellulose as vehicle. After 2 months of treatment, paraffin embedded cerebral cortex sections were subjected to periodic acid-Schiff (PAS) stain for analyzing glycolipids. The glycoconjugate material stain magenta shown by the arrows (A) was quantified by PAS +ve cells per mm square (B) in 10 sections per group. Paraffin sections were stained for H & E staining followed by characterization of large vacuoles (arrows), pyknotic nuclei and swollen neuron (C). Number of vacuoles were quantified per mm square (D) in 9 sections per group. Data represents mean ± SEM. Statistical analysis was performed by one-way ANOVA followed by Dunnett’s multiple comparison test. ^***^p<0.001.

Given the observation that CA can activate PPARα [22], we explored the hypothesis that activation of PPARα by CA could be the underlying mechanism by which it exhibits the glycoconjugate lowering effect. We compared the glycoconjugate pathology of the motor cortex region among all the groups *viz*. NTg, Sandhoff, Sandhoff-treated with CA, Sandhoff^ΔPPARα^, and Sandhoff^ΔPPARα^ -treated with CA using PAS stain followed by quantification of PAS +ve granules per mm square (Figure 7A and B). Further, Hematoxylin & Eosin staining was done followed by quantification of number of vacuoles in the similar groups (Figure 7C and D). Histological observation using PAS stain revealed increased glycoconjugate storage material in both Sandhoff and Sandhoff^ΔPPARα^ mice when compared to normal NTg mice (Figure 7A and B). However, treatment with CA25 led to significant clearance of storage materials from the cerebral cortex of Sandhoff mice but there was no alteration in the levels of storage materials in CA25-treated Sandhoff^ΔPPARα^ null mice (Figure 7A and B). This suggests that cinnamic acid requires PPARα for its glycoconjugate-mitigating effects. Similarly, routine H&E analysis of the same groups revealed significant increase in neuronal vacuolation in both Sandhoff and Sandhoff^ΔPPARα^ as compared to normal NTg (Figure 7C and D). Histologic examination revealed neurons to be of balloon shaped with cytoplasmic vacuoles (Figure 7C and D). On the other hand, Sandhoff mice treated with CA25 showed substantial reduction in vacuolation but there was no change in the CA25-treated Sandhoff^ΔPPARα^ mice (Figure 7C and D). Together, our findings suggest that cinnamic acid treatment of Sandhoff mice could mitigate glycoconjugate pathology in a PPARα-dependent manner.

### Oral administration of CA improves motor deficits and increases survival in Sandhoff mice via PPARα

**Figure 8:**
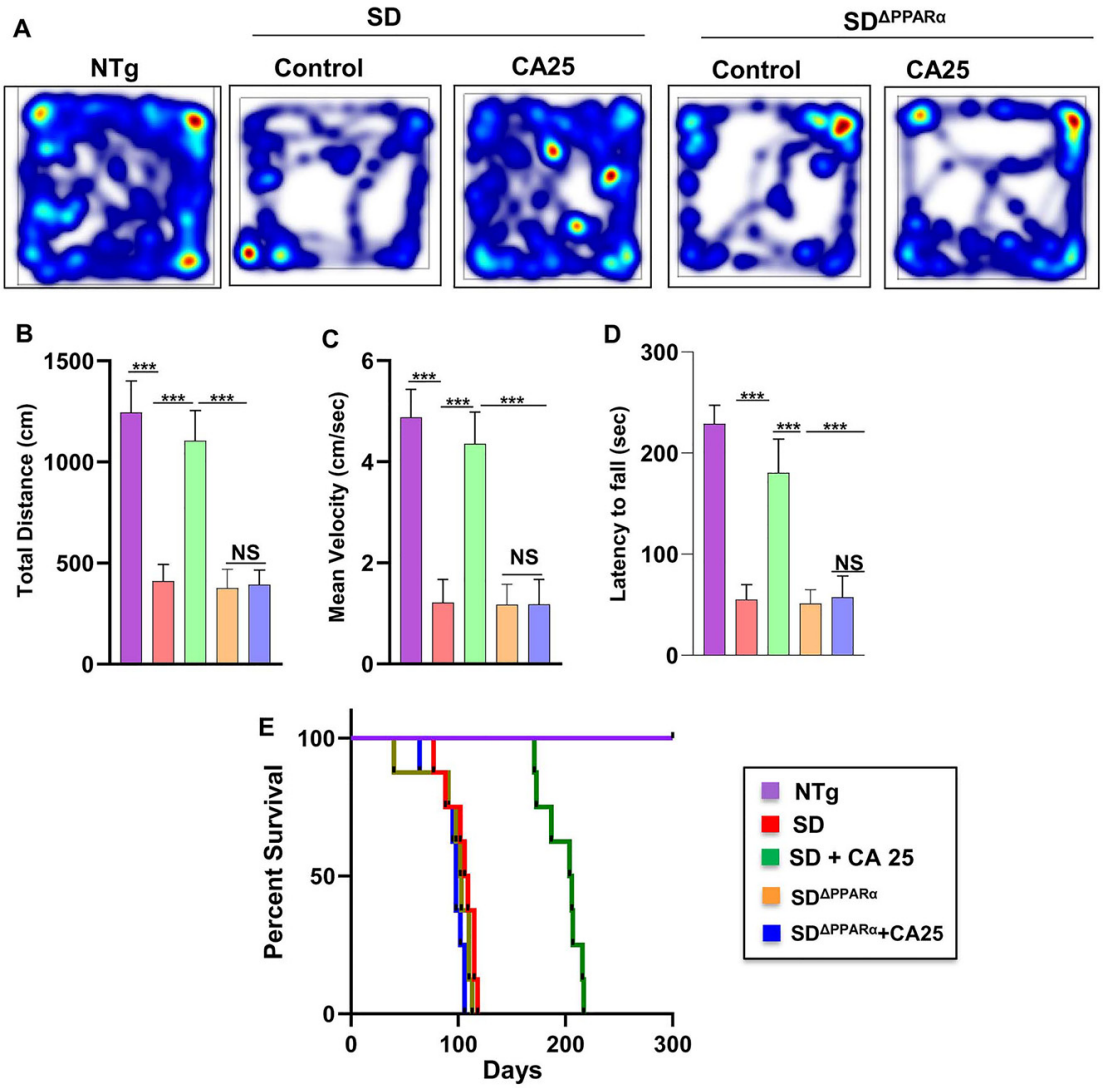
CA administration reduces motor deficits and increases survival in Sandhoff mice via PPARα. Three-months-old SD and SD^ΔPPARα^ mice (n=6/group) were treated orally with CA (25 mg/kg/day) solubilized in 100 µL 0.5 % methylcellulose via gavage. After 2 months of treatment, horizontal locomotor activities of experimental animals were monitored in the open field arena by the Noldus software (A, heatmap; B, total distance; C, mean velocity). Latency of each mouse to fall on the base of a rotarod was monitored (D). Kaplan–Meier survival analysis (n=8) (E). Data represent mean ± SEM. Statistical analysis was performed by one-way ANOVA followed by Dunnett’s multiple comparison test; ^***^p<0.001; ns, not significant.

Finally, we analyzed the effect of CA on the behavioral performance of NTg, Sandhoff, CA25-treated Sandhoff, Sandhoff^ΔPPARα^, and CA25-treated Sandhoff^ΔPPARα^ mice. We examined the general locomotor activity of these mice in the open field test (Figure 8A), which showed significant differences in the total distance traveled (Figure 8B) and mean velocity (Figure 8C) of different cohorts of mice. Sandhoff mice and Sandhoff^ΔPPARα^ mice demonstrated severe impairment in open field test as shown by reduction in movement (Figure 8A), total distance (Figure 8B) and velocity (Figure 8C). The Sandhoff and Sandhoff^ΔPPARα^ mice also exhibited poor performance in rotarod test (Figure 8D), affirming the compromised motor cortex parameters in these groups of mice. However, CA25 treatment reversed the poor motor performance & locomotor activities of the Sandhoff animals, but not the Sandhoff^ΔPPARα^ mice, indicating that oral administration of CA improves motor deficits in Sandhoff mice via PPARα.

Survival curves for each treatment group *viz.* NTg, Sandhoff, CA25-treated Sandhoff, Sandhoff^ΔPPARα^, and CA25-treated Sandhoff^ΔPPARα^ mice were estimated. Interestingly, CA25-treated Sandhoff mice survived more than 6 months as shown by the Kaplan Meier survival curve analysis (Figure 8E). However, CA25-treated Sandhoff^ΔPPARα^ mice failed to survive beyond 3 months quite similar to either Sandhoff or Sandhoff^ΔPPARα^ mice (Figure 8E), suggesting an essential role of PPARα in cinnamic acid-mediated increased longevity of Sandhoff mice.

## Discussion

Tay-Sachs disease (TSD) and Sandhoff disease (SD) are progressive and autosomal recessive neurodegenerative disorder, resulting in abnormal accumulation of GM2 gangliosides or glycosphingolipids [38]. Infants with SD develop symptoms like loss of motor function and cognition, developmental regression, hind limb plasticity, muscle weakness, dystonia, blindness, seizures, and eventually death in childhood [39]. In spite of intense investigations, there is no effective therapy or lines of treatment available for these diseases. Gene therapy has been proposed to restore deficient enzymes in the brain, however, it is extremely costly. Moreover, approaches with adeno-associated viral vectors (AAVs) still pose safety and efficacy issues, thereby supporting the search for alternative therapeutic strategies. Apart from gene therapy, several other therapeutic approaches have been recommended in the treatment regime. The most common ones are therapeutic enzyme replacement [40], bone marrow transplantation [41], substrate deprivation therapy [42], hematopoietic [43] or neural stem cell transplantation [44] use of chemical chaperones and oligonucleotide recombination [45], or a combination of all. However, none of these therapies has led to a successful treatment option for these diseases. Cinnamic acid (CA), a naturally occurring plant-based product, is available in different vegetables, fruits, honey, and whole grains [20, 46]. Moreover, it is also a component of cinnamon, a commonly used spice and a flavoring additive in a wide variety of foods [47]. Several studies have highlighted the role of CA and its pharmacologically active derivatives as anti-microbial, anti-oxidant, anti-cancer, anti-atherogenic, and anti-fungal agents [48], [49], [50], [51]. Our lab has shown that oral CA is capable of reducing amyloid plaques from the brain of an animal model of AD [22] and protecting nigral dopaminergic neurons in an animal model of PD [52]. Here, we delineate that CA also exhibits neuroprotection in an animal model of SD. Two months of CA treatment markedly decreased microglial and astroglial activation in the cerebral cortex of Sandhoff mice. We also observed improved gait and behavioral performances of Sandhoff mice after CA treatment. Moreover, significant increase in life expectancy of Sandhoff mice was found after oral CA treatment. Therefore, oral CA may be beneficial for SD.

One of the hallmarks of SD is the accumulation of G_M2_ ganglioside in the CNS, indicating that successful treatments should exhibit clearance of ganglioside deposition from the brain. It is nice to see that CA treatment markedly reduced ganglioside deposition from the cerebral cortex of Sandhoff mice. Consistent to the fact that the accumulation of undegraded gangliosides is associated to the activation of microglial cells [53, 54], CA treatment reduced microglial inflammation *in vivo* in the brain of Sandhoff mice. It has been shown that pathological accumulation of GM1 or GM2 is capable of triggering apoptosis [55], [56], [57]. Accordingly, although we found marked apoptosis in the cerebral cortex of Sandhoff mice, it was strongly suppressed by CA treatment. Together, oral CA treatment decreased glial inflammation and protected neurons from apoptosis probably via clearance of pathological ganglioside accumulation.

Mechanisms by which CA may reduce ganglioside accumulation from the brain are poorly understood. Autophagy plays an important role in the removal of any biomolecule accumulation from any tissues and organs. According to Chandra et al. [22], CA stimulates lysosomal biogenesis and autophagy in brain cells. Transcription factor EB (TFEB) is considered as the master regulator of lysosomal biogenesis and autophagy [34, 58]. It has been found that the *TFEB* gene promoter harbors a peroxisome proliferator response element and that activation of peroxisome proliferator-activated receptor α (PPARα) stimulates the transcription of *TFEB* gene via PPRE [35, 59], [60], [61]. Although the classical role of PPARα is to stimulate the biogenesis of peroxisomes [37, 62], we have seen that PPARα is also responsible for lysosomal biogenesis [35, 59]. Interestingly, Chandra et al. [22] have also reported that CA is capable of binding to the ligand-binding domain of PPARα and activating this transcription factor. Moreover, activation of PPARα has also been reported to reduce neuroinflammation via upregulation of anti-inflammatory molecules such as SOCS3 and IL-1Ra [63, 64]. In the current work, we found decrease in PPARα in the brains of Sandhoff mice as compared to non-transgenic mice. Consistent to the upregulation of PPARα by CA [22], oral administration of CA increased the level of PPARα *in vivo* in the cerebral cortex of Sandhoff mice. Hence, to find out the core mechanism behind ganglioside reduction and neuroprotection, we prepared Sandhoff^ΔPPARα^ mice in which CA remained unable to reduce the accumulation of PAS stained glycoconjugates and improve life expectancy. Therefore, it appears that oral CA is exhibiting anti-inflammation in the brain, lowering the load of gangliosides from the brain and increasing longevity of Sandhoff mice via PPARα.

There are several advantages of CA over other proposed anti-neuroinflammatory and anti-neurodegenerative therapies for SD because CA is a natural plant-based product and is relatively non-toxic. It can be taken orally, the least painful route. It is present in fruits, vegetables and grains that are consumed by people throughout the world on a regular basis. Moreover, CA is readily available, economical and nontoxic as compared to other available SD therapeutics.

In summary, our study demonstrates that oral CA treatment attenuates glial inflammation, reduces glycoconjugate accumulation, decreases neuronal apoptosis, improves gait & locomotor activities, and increases survival in a mouse model of Sandhoff disease through PPARα. Therefore, CA may have therapeutic implications for lowering the pathogenesis of SD.

## Supplementary Material

Supplementary Material Details
