# Supplementary material for: Cinnamic acid, a natural plant compound, exhibits neuroprotection in a mouse model of Sandhoff disease via PPARα
Source: NeuroImmune Pharm Ther. 2024 Mar 15;3(1):17–32. doi: 10.1515/nipt-2023-0027 (PMC10961485; doi:10.1515/nipt-2023-0027)
Supplement: Supplementary file 1 — Supplementary Material Details [file j_nipt-2023-0027_suppl_001.pdf]

Cinnamic acid, a natural plant compound, exhibits neuroprotection in mouse model of Sandhoff disease via PPARα

Sumita Raha[1], Ramesh K. Paidi[1], Debashis Dutta[1] and Kalipada Pahan[1,2]

[1]Department of Neurological Sciences, Rush University Medical Center, Chicago, IL, USA;
[2]Division of Research and Development, Jesse Brown Veterans Affairs Medical Center, Chicago, IL, USA

Running title: Cinnamic acid and GM2 gangliosidosis

Address correspondence:
Kalipada Pahan, Ph.D.
Department of Neurological Sciences
Rush University Medical Center
1735 West Harrison St, Suite Cohn 310
Chicago, IL 60612,
Tel: (312) 563-3592
Fax: (312) 563-3571
Email: Kalipada_Pahan@rush.edu

Supplemental results

Legends to supplemental figures:

Figure S1. Raw blots for Figure 1F.

Figure S2. Raw blots for Figure 2D.

Figure S3. Raw blots for Figure 6D.

Figure 1F

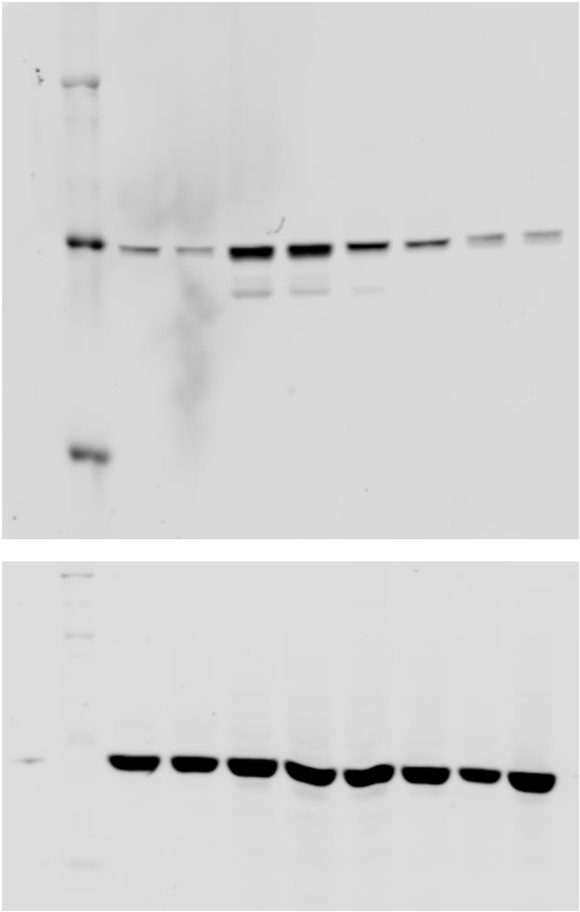

Figure S1. Raw blots for Figure 1F

Figure 2D

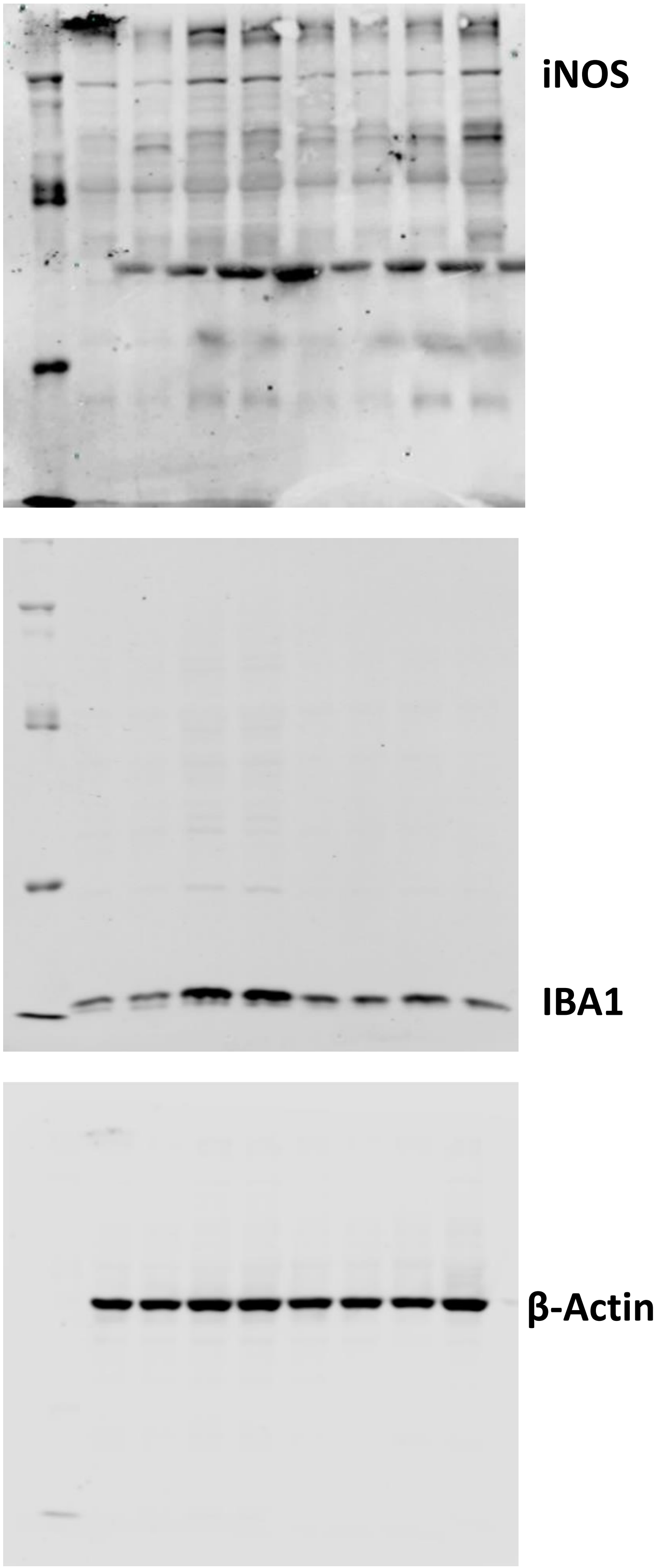

Figure S2. Raw blots for Figure 2D

Figure 6D

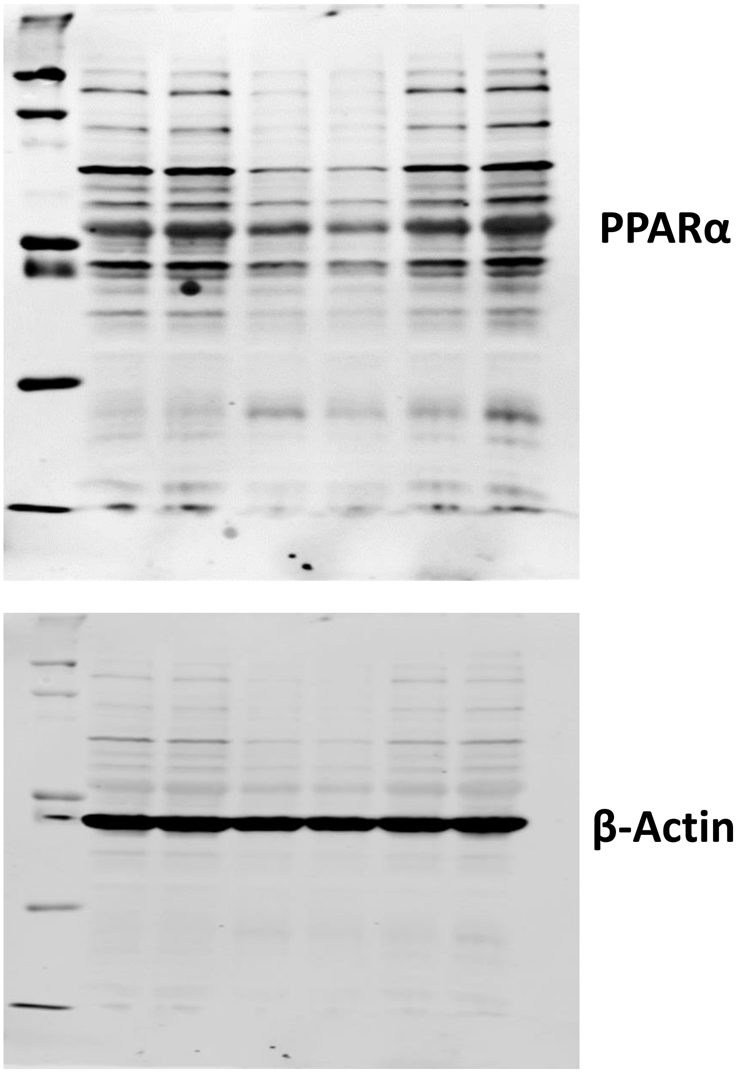

Figure S3. Raw blots for Figure 6D
